# A recurrent side-changing febrile pleural effusion revealing familial Mediterranean fever: a case report

**DOI:** 10.11604/pamj.2022.43.121.33324

**Published:** 2022-11-03

**Authors:** Islam Mejri, Asma Saidane, Nouha Boubaker, Samira Mhamdi, Selsabil Daboussi, Chiraz Aichaouia, Zied Moatemri

**Affiliations:** 1 Military Hospital, Department of Pulmonology, Tunis, Tunisia,; 2 University of Tunis El Manar, Faculty of Medicine of Tunis, 1007 Tunis, Tunisia

**Keywords:** Familial Mediterranean fever, fever, pleural effusion, familial Mediterranean fever gene, case report

## Abstract

Familial Mediterranean Fever (FMF), characterized by recurrent polyserositis, is an autosomal recessive disease involving essentially Mediterranean populations. We report the case of a 30-year-old Tunisian military patient complaining of fever and chest pain recurring on board a Navy military vessel, due to side-changing pleural effusion. On landing, a marked improvement of symptoms was noticed. Gene testing was performed when the diagnostic survey ruled out common etiologies, revealing a homozygous mutation of the FMF gene type M680l/M680l. The prescription of colchicine and the exemption from boarding led to the resolution of the symptoms with no recurrence of pleural effusion. Therefore, the diagnosis of FMF should be considered in a context of a recurrent pleural effusion in the youth, with a negative etiological assessment, notably in an ethnic group at risk. Thus, early diagnosis and adequate treatment may prevent the development of secondary amyloidosis, a serious complication of FMF.

## Introduction

Familial Mediterranean Fever (FMF) is the most common inherited auto-inflammatory disease [1]. It results in recurrent acute attacks of febrile polyserositis (peritonitis, arthritis, pleurisy…). Pleural effusion and fever are the only symptoms of the disease in 5-10% of patients [2]. We report a case of recurrent, febrile, self-resolving pleural effusion in a Tunisian military Navy officer confirmed by a genetic screen.

## Patient and observation

**Patient information:** a 30-year-old nonsmoker patient was referred to our pulmonology department. He was operating in the Tunisian marine Navy with no significant personal or familial past medical history. He complained of left-sided chest pain having started 10 days ago, with dyspnea on exertion and diffuse arthralgia. A similar episode occurred six months ago on board the Navy vessel, treated empirically with antibiotics and corticosteroids.

**Figure 1:**
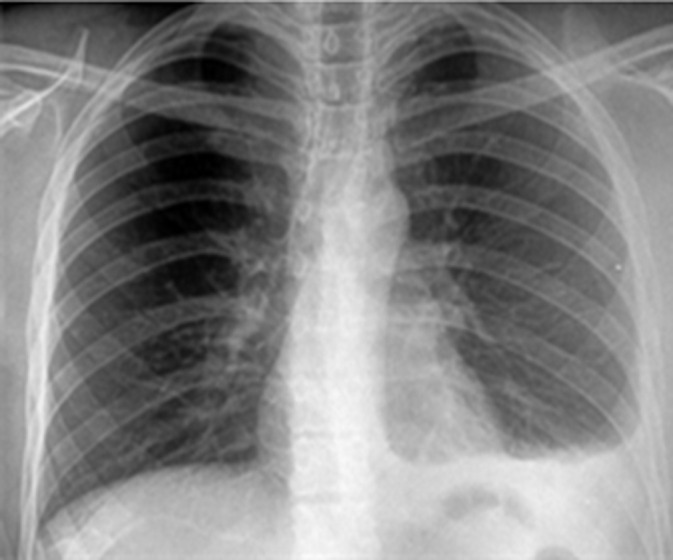
chest radiography showing minimal left-sided pleural effusion

**Figure 2:**
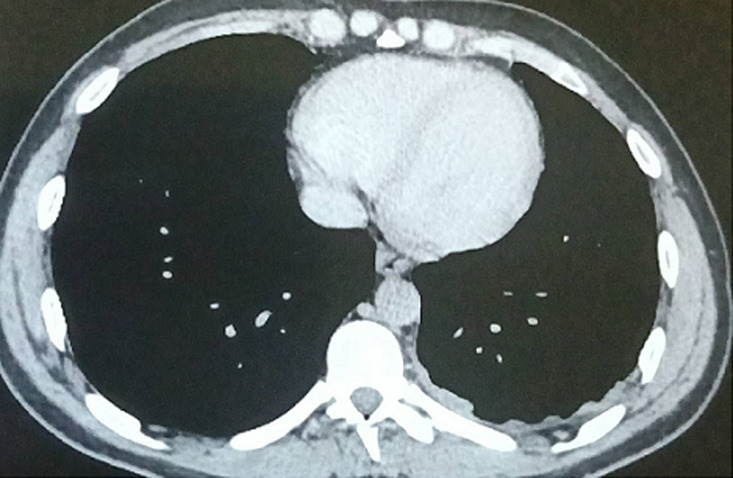
thorax computed tomography showing left-sided pleural thickening

**Clinical findings:** physical examination was normal except for left-sided pleuretic syndrome. The chest X-ray revealed a minimal left-sided pleural effusion (Figure 1). The contrast-enhanced chest-computer tomography showed a mild pleural fluid in the left chest, without any parenchymal involvement, and ruled out embolism (Figure 2). Nevertheless, a pleural puncture or biopsy could not be performed because of the rapid regression of the effusion.

**Figure 3:**
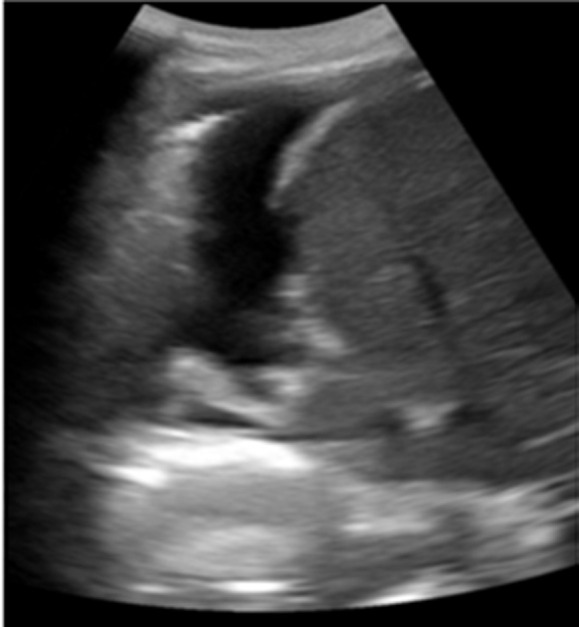
ultrasound showing minimal right-sided pleural effusion

**Timeline of current episode:** at the time, the patient had received an antibiotic (Amoxicillin-clavulanic acid) without any proof of infection for 7 days. After a partial clinical and radiological improvement, he was discharged. Four months later, he was admitted for recurrence of the chest pain on the contralateral side, with vomiting and mild fever (+38°C). No abdominal pain was reported. The chest X-Ray showed a minimal right-sided pleural effusion. The chest ultrasound confirmed the presence of a minimal right-sided hypoechoic unloculated pleurisy (Figure 3).

**Diagnostic assessment:** laboratory investigations revealed a leukocytosis (10400/mm^3^) and an elevated C-reactive protein (CRP) (221 mg/l). The renal test didn't show any microscopic hematuria or proteinuria. The tuberculin skin test and immunological screen were negative. Serum protein electrophoresis was normal. The echocardiography and abdominal ultrasound were normal.

**Diagnosis:** the diagnosis of familial Mediterranean fever was considered by, in light of: recurrent, febrile, self-resolving episodes of a side-changing pleural effusion and arthralgia, without any underlying cause that could explain these attacks. The genetic testing showed a homozygous mutation of the Mediterranean Fever (MFEV) gene type (M680I/M680I).

**Therapeutic interventions:** colchicine was initiated at a dosage of 1 mg daily with an immediate improvement. Besides, we referred our patient to an occupational physician for professional redeployment, to prevent the recurrence of further attacks. Screening the family members identified similar asymptomatic cases in the brotherhood.

**Follow-up and outcome of interventions:** six months and one year later, he was seen on routine follow-up. He was asymptomatic, without any evidence of pleural or pericardial effusion.

**Patient perspective:** “I feel relieved since I have a diagnosis for my symptoms. Currently, I know how to manage my illness and relieve my pain”. “Since I have been on medication, I have a better quality of life with no pain or discomfort”. “I am now sensitizing my family to screen this disease”.

**Informed consent:** written consent was obtained from the patient to publish images and clinical information relating to the case. The patient consented to his clinical information except name to be published to contribute to science and global health.

## Discussion

Familial Mediterranean fever is an auto-inflammatory autosomal recessive disease, affecting mainly people of Mediterranean ancestry (Sephardic Jews, Arabs, Armenian…). The main locations of the disease are (the peritoneum, the synovium, the joints...). However, the pleural or the pericardial involvement remains uncommon [3]. We report a case of a Tunisian marine officer diagnosed with FMF revealed by recurrent febrile episodes of side-changing pleural effusion. The diagnosis was challenging in our case, especially in the absence of a positive family history of FMF or consanguinity. This disease may be responsible for a wide spectrum of symptoms (chest pain, fever, arthralgia…). Our study case complained of fever, chest pain, and dyspnea. Acute attacks may be triggered by many factors: infections, stress, intense physical activity, extreme variation of temperatures and menstruation [4]. Our patient has worked as a marine for years in a Mediterranean region (Tunisia). Extreme climatic changes and hard physical activity could have precipitated these acute episodes. Laboratory tests may show an increased level of acute inflammation proteins (CRP, Fibrinogen, and Interleukin 6…). We did not find any underlying infection in our case that could explain these recurrent attacks. Imaging may objectify pleural thickening or minimal pleural plaques due to the recurrence of acute inflammatory attacks. The pleural biopsy may show a chronic, non-specific inflammation. However, it was not performed in our case because of the small amount of the effusion and its rapid regression.

Familial Mediterranean fever, also known as «recurrent polyserositis» or «the periodic disease» is a genetic condition due to a mutation of the MFEV gene located in the short arm of the chromosome 16 which encodes for a regulatory protein called «Pyrin», involved in the systemic inflammatory process [5]. Recent studies suggest that genetic haplotypes may have a prognostic value [6]. In our case, the mutation of MFEV gene, at exon 10 type (M680I/M680I), is known to be associated with a greater risk of secondary amyloidosis and worse outcomes. Besides, Federici *et al*. have suggested that patients with 2-high homozygous mutation alleles (M680I) develop the disease earlier and are quite more symptomatic with more severe complications as well, than those with one mutation allele (M680I) [7].

Genetic testing may be useful in such atypical cases. However, it is not mandatory. In fact, the FMF is a clinical condition. Many scores have been found to help clinicians assess the diagnosis of FMF, such as the «Tel-Hashomer diagnostic criteria» [8]. Global management aims to prevent further attacks and the silent deposition of Amyloid substances. Colchicine is the mainstream of the treatment. It should be given orally at a dosage of (1-2mg daily). Our patient has completely responded to an adequate dose of Colchicine therapy. In case of resistance, numerous treatments have been tested such as Immunosuppressive drugs (TNF-Alpha Inhibitor, Thalidomid…) and Immunomodulators (Interleukin1 Beta Inhibitor, Etanercept…) [9]. Further researches are required to better understand the underlying mechanisms of this disease and to find out new insights for adequate treatments.

## Conclusion

This is a rare case of FMF revealed by recurrent pleural effusion confirmed by genetic testing. The early diagnosis led to the relief of symptoms in our patient and to reveal undiagnosed new cases in his family. Our case report highlights the importance of early screening and treatment of this disease, in order to prevent further attacks and the development of secondary amyloidosis.
